# Blood cytokine levels in anti-NMDAR and viral encephalitis during the early stage of hospitalization: a small-sample exploratory pilot study

**DOI:** 10.3389/fimmu.2026.1763289

**Published:** 2026-04-01

**Authors:** Yingshi Guo, Yingzhu Zhao, Yujie Yi, Hong Zhou, Kaiye Xing, Ying Yu, Jie Wang

**Affiliations:** 1 First Clinical Medical College of Shanxi Medical University, Taiyuan, China; 2 Department of Neurology, First Hospital of Shanxi Medical University, Taiyuan, China

**Keywords:** anti-NMDAR encephalitis, CSF/serum albumin ratio, cytokines, exploratory pilot study, virus encephalitis

## Abstract

**Objectives:**

We aimed to perform a preliminary analysis of early cytokine profiles in blood between patients with anti-N-methyl-D-aspartate receptor (anti-NMDAR) encephalitis and viral encephalitis (VE), and to observe potential preliminary trends.

**Methods:**

This study adopted a retrospective design. Following a standardized screening process, anti-NMDAR encephalitis (n = 15), VE (n = 15), and non-inflammatory central nervous system diseases as controls (n = 15) were retrospectively enrolled. Correlations between candidate biomarkers and clinical characteristics of two kinds of encephalitis patients were analyzed.

**Results:**

This study identified significant alterations in the CSF/serum albumin ratio (QAlb), IL-4 and IL-17A levels across among the two patient groups and controls. These markers exhibited certain diagnostic potential for differentiating anti-NMDAR encephalitis from VE. In the anti-NMDAR encephalitis group, IL-17A and QAlb were positively correlated with the patients’ hospital stay duration and mRS score at admission (P<0.05). In the VE group, IL-4 and QAlb were associated with the patients’ hospital stay duration and disease severity (P<0.05), with IL-4 showing a negative correlation. However, the present findings should be regarded as preliminary and require further confirmation before clinical translation.

**Conclusions:**

Elevated levels of specific inflammatory indicators showed potential as candidate biomarkers to aid in the early differential diagnosis between anti-NMDAR encephalitis and VE.

## 1 Introduction

Anti-N-methyl-D-aspartate receptor (anti-NMDAR) encephalitis is an autoimmune disorder mediated by anti-NMDAR antibodies, representing one of the most common forms of autoimmune encephalitis (AE) in clinical practice (1). This disease can lead to a range of complex neurological and psychiatric symptoms, and in severe cases, may become life-threatening (2, 3). Early diagnosis and prompt initiation of immunotherapy have been shown to significantly improve patient outcomes (4, 5). Viral encephalitis (VE), on the other hand, results from viral infections and often presents with clinical features and routine cerebrospinal fluid (CSF) findings that overlap considerably with those of anti-NMDAR encephalitis (4). As a result, differentiating between these two entities in the early stages remains a considerable clinical challenge. The key diagnostic distinctions rely primarily on the detection of specific antibodies in the CSF and identification of viral pathogens. However, these tests are time-consuming, susceptible to false-negative results, and often exhibit low positive rates, which can contribute to misdiagnosis or delayed diagnosis. Therefore, there is a critical need to identify readily available and early predictive biomarkers that can facilitate timely differentiation between anti-NMDAR encephalitis and VE. Such markers would support earlier intervention, guide appropriate treatment strategies, and ultimately improve patient prognoses.

CSF/serum albumin ratio (QAlb, the ratio of albumin concentration in cerebrospinal fluid to that in serum) reflects the integrity of the blood-brain barrier (BBB). In clinical practice, the integrity of brain barriers cannot be evaluated directly by visualization. BBB impairment is a key pathophysiological feature in many central nervous system (CNS) disorders, including epilepsy, Alzheimer’s disease, and multiple sclerosis (6–8). QAlb is currently recognized as the easiest and most reliable biomarker for evaluating the permeability of brain barriers, including blood–brain barrier and blood-CSF barrier (9).

Neutrophil-to-lymphocyte ratio (NLR, the ratio of neutrophil count to lymphocyte count) is a common inflammatory marker. It has demonstrated prognostic value in predicting severity and mortality in certain cancers and cardiovascular conditions (10, 11). Previous studies have also established NLR as a reliable marker for monitoring disease activity in CNS demyelinating disorders, predicting hemorrhagic transformation after stroke, and assessing clinical outcomes in acute embolic event (12–14).

Cytokines are frequently utilized as indicators to ascertain whether the body is infected (15). Furthermore, an increase in cytokines has been demonstrated to induce a pro-inflammatory effect (16). Studies have shown distinct cytokine profiles in anti-NMDAR encephalitis and VE, suggesting differences in their underlying immune mechanisms (17–19). Commonly measured serum cytokines in clinical practice include interleukin IL-2, IL-4, IL-6, IL-10, IL-17A, tumor necrosis factor (TNF)-α, and interferon (IFN)-γ.

Cytokines, QAlb, and NLR are widely used clinical biomarkers. In certain neurological disorders, there have been isolated reports on the expression of specific cytokines in the central nervous system. Nevertheless, studies that utilize peripheral blood as a rapid and non-invasive screening tool remain comparatively infrequent (20, 21). It remains unclear whether these biomarkers exhibit differential expression in the early stages of anti-NMDAR encephalitis compared to VE, warranting further investigation. The present study aims to identify and evaluate commonly available, easily measurable biomarkers—including serum cytokines, QAlb, and NLR—by analyzing their differential expression patterns in patients with anti-NMDAR encephalitis versus VE. Furthermore, we seek to pinpoint discriminative indicators and explore their association with disease severity. The findings are expected to facilitate the identification of practical biomarkers and establish a basis for early clinical discrimination between anti-NMDAR encephalitis and VE.

## 2 Materials and methods

### 2.1 Patients

We retrospectively examined the medical records of all patients diagnosed with anti-NMDAR encephalitis and VE and admitted to the First Hospital of Shanxi Medical University between January 2021 to October 2023. All of the patients had received neurologic evaluation, magnetic resonance imaging, blood collection and its laboratory tests such as cytokines, QAlb, and NLR within 72 hours of admission, prior to the administration of any immunotherapy or anti-infection treatment. All diagnoses were established by experienced neurologists according to standard clinical and laboratory criteria.

#### 2.1.1 anti-NMDAR encephalitis group

A retrospective study was conducted on 132 patients diagnosed with AE. 37 patients were diagnosed with anti-NMDAR encephalitis based on positive anti-NMDAR antibody in CSF or serum, combined with compatible clinical manifestations and typical radiological features, according to internationally recognized diagnostic criteria (22). Finally, 15 patients were enrolled in the study, with the following inclusion criteria: (1) age ≥ 18 years and survival during hospitalization; (2) no antibodies against other neuronal antigens detected in both serum and CSF; (3) complete blood sample collection and analysis performed prior to immunotherapy.

#### 2.1.2 VE group

During the same period, 153 patients diagnosed with VE (23). Twenty-one patients with unknown pathogens or mixed infections were excluded. Finally, 49 patients were enrolled in the study, with the following inclusion criteria: (1) age ≥18 years; (2) viral infection in CSF confirmed by metagenomic next-generation sequencing (mNGS), ELISA, or PCR; (3) presence of symptoms indicating brain parenchyma damage, such as fever, headache, vomiting, convulsion, mental abnormality, and disturbance of consciousness; (4) a marked improvement in the condition was observed following the administration of anti-infection treatment. Subsequently, stratified random sampling was performed among the eligible patients using SPSS 26.0 software, stratified by age (18–40 years, 41–60 years, and >60 years) and gender. A total of 15 patients were selected to form the viral encephalitis group.

#### 2.1.3 Control group

Control patients were recruited during the same period from the same department, admitted for acute neurological symptoms. To ensure comparability with the anti-NMDAR encephalitis group and viral encephalitis group, the age- and sex-matched individuals with noninflammatory diseases served as controls. The control group met the following criteria: (1) there were no obvious abnormalities in CSF cytology examination; (2) there were no definite or suspicious CNS infectious diseases; (3) there were no definite or suspected neuromyelitis optica spectrum disorder or multiple sclerosis; and (4) there were no definite or suspicious systemic autoimmune diseases. Patients with hydrocephalus, benign intracranial hypertension and venous sinus thrombosis were mainly included.

### 2.2 Clinical data

Basic clinical data collected included age, gender, mental status, epileptic seizures, central hypoventilation, fever, and level of consciousness, along with tumor-related information. Laboratory parameters, including serum cytokine levels, the NLR, and the QAlb, were also obtained for analysis. Disease severity was assessed using the modified Rankin Scale (mRS) score on admission and hospital length of stay. All the information had been collected within 72 hours of admission, prior to the administration of any immunotherapy or anti-infection treatment. And all data were collected in a standardized manner from electronic medical records.

### 2.3 Statistical analysis

Statistical analysis was conducted using SPSS 26.0, and graphs were created with GraphPad Prism 10.1.2. The Shapiro-Wilk test was used to check for normality of the data. For normally distributed data, the mean ± standard deviation is used; otherwise, the median (maximum value, minimum value) is used. Quantitative data are presented as number (percentage), and qualitative data are presented as rate. The one-way analysis of variance (ANOVA) with Bonferroni-corrected *post-hoc* tests was performed to compare the levels of cytokines and inflammatory mediators among the three groups. For data that follow a normal distribution, Pearson’s correlation is used for line correlation analysis; otherwise, Spearman’s correlation is used. To evaluate the performance of factors in differentiating anti-NMDAR encephalitis from VE and determine the threshold values, the receiver operating characteristic (ROC) analyses were used. Cutoff points for factors with diagnostic value were determined by likelihood ratio positive = sensitivity/(1 − specificity) from described ROC curves. P <0.05 indicates statistical significance.

## 3 Results

### 3.1 Baseline and demographic features

**Figure 1:**
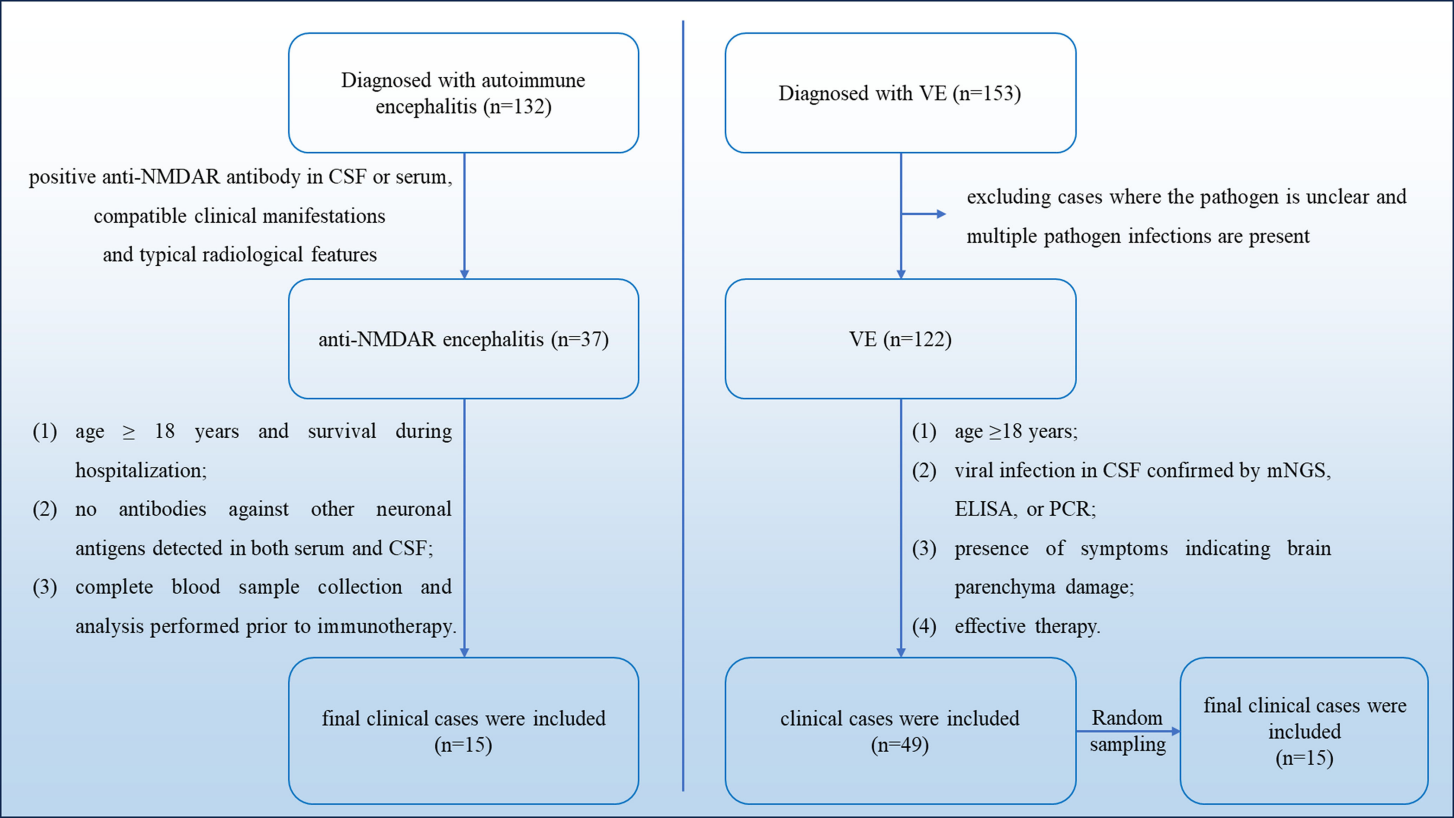
Selection process about clinical data.

According to strict selection criteria, 15 patients were selected from each group (**Figure 1**). The ages, genders and clinical manifestations were statistically analyzed (**Table 1**). The results showed that there was no statistical difference between the two groups of encephalitis in terms of age, gender and clinical symptoms (p > 0.05). At the same time, to verify the comparability of the included and excluded VE, we conducted a baseline balance analysis of key demographic and clinical indicators. The results showed that the baseline characteristics of the two groups were balanced and comparable (p > 0.05) (**Supplementary Table 1**).

**Table 1:** Demographics and baseline clinical characteristics.

Baseline characteristics	Anti-NMDAR encephalitis (n=15)	VE (n=15)	Control (n=15)	P-value (two groups of encephalitis)
Age	38.33 ± 17.66	44.73 ± 18.21	51.07 ± 14.25	0.337 ^a^
Sex(Female)	11(73.33%)	10(66.67%)	5(33.33%)	1.000 ^b^
Mental symptom	11(73.33%)	10(66.67%)	2(13.33%)	1.000 ^b^
Epilepsy	8(53.33%)	7(46.67%)	0(0.00%)	0.715 ^b^
Central hypoventilation	6(40.00%)	4(26.67%)	0(0.00%)	0.439 ^b^
Fever	2(13.33%)	5(33.33%)	0(0.00%)	0.388 ^b^
Lowering of consciousness	10(66.66%)	8(53.33%)	0(0.00%)	0.456 ^b^
Tumor	2(13.33%)	0(0.00%)	0(0.00%)	0.464 ^b^

^a^ Student’s t-test; ^b^ Chi-Square test. Data are presented as mean ± SD for normally distributed continuous variables, and as median (maximum, minimum) for non-normally distributed continuous. The enumeration data are n (%).

anti-NMDAR, anti-N-methyl-D-aspartate receptor; VE, viral encephalitis.

### 3.2 Indicator characteristics

We collected data on patient serum cytokines (IL-2, IL-6, IL-4, IL-10, IL-17A, TNF-α, IFN-γ), QAlb, and NLR. The results showed that the expressions of IL-4, IL-6, IL-17A and QAlb were higher in two encephalitis groups than control group, and there were significant differences (p < 0.05) (**Table 2**).

**Table 2:** Comparison of nine cytokines and inflammatory mediator levels in the blood among anti-NMDAR encephalitis, VE and controls.

Indicators	Anti-NMDAR encephalitis (N = 15)	VE (N = 15)	Control (N = 15)	*F* value	P-value
IL2 (pg/ml)	1.31 ± 0.90	1.36 ± 0.99	0.68 ± 0.94	0.709	0.498
IL4 (pg/ml)	1.90 ± 1.50	2.80 ± 0.84	0.98 ± 0.37	26.717	<0.001
IL6 (pg/ml)	7.10 ± 9.39	13.09 ± 8.58	4.05 ± 2.09	4.930	0.012
IL10 (pg/ml)	2.46 ± 1.70	2.46 ± 2.25	2.60 ± 2.86	1.898	0.163
IL17A (pg/ml)	1.90 ± 7.56	1.02 ± 1.05	0.02 ± 0.37	8.504	0.001
IFN-γ (pg/ml)	1.00 ± 0.73	1.10 ± 0.78	0.52 ± 1.73	0.831	0.443
TNF-α (pg/ml)	1.21 ± 1.21	1.39 ± 0.99	1.48 ± 1.55	0.029	0.971
QAlb	11.06 ± 5.85	17.23 ± 17.03	6.98 ± 1.74	15.868	<0.001
NLR	3.03 ± 1.14	3.88 ± 2.90	1.59 ± 1.00	3.119	0.055

Data are presented as mean ± SD. P-value: one-way ANOVA.

anti-NMDAR, anti-N-methyl-D-aspartate receptor; VE, viral encephalitis; TNF, tumor necrosis factor; IFN, interferon; NLR, the neutrophil-to-lymphocyte ratio; QAlb, the CSF/serum albumin ratio.

**Figure 2:**
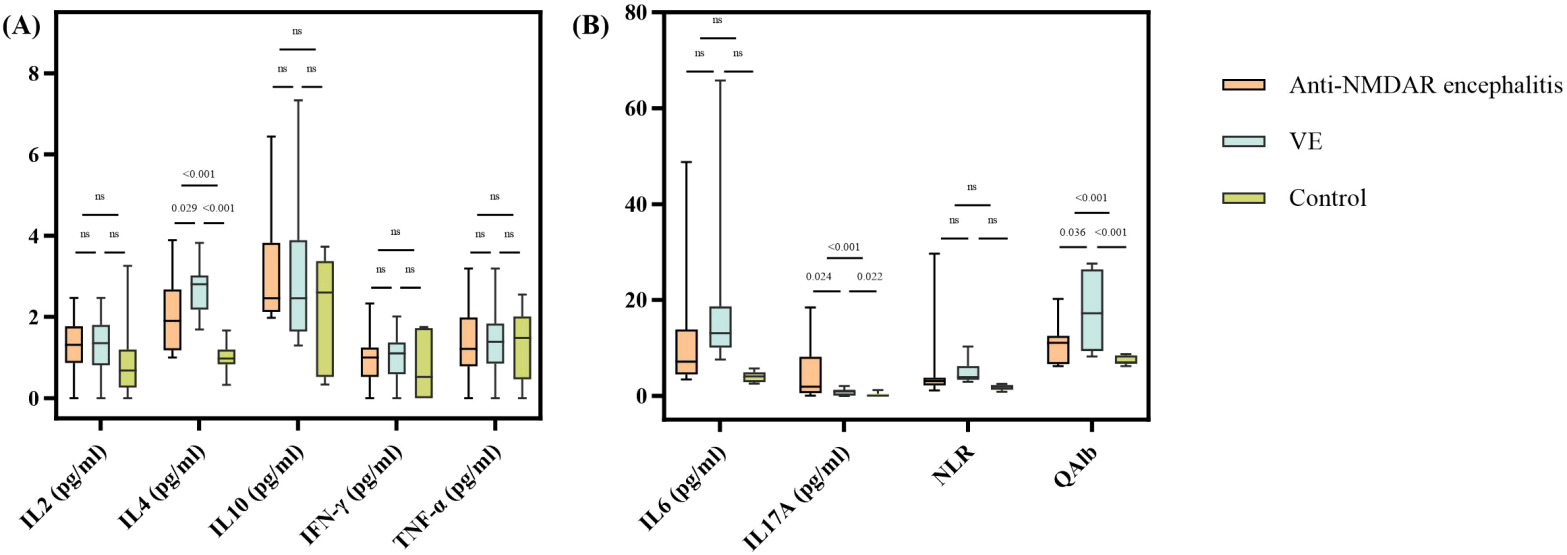
Differential analysis of serum cytokines (IL-2, IL-4, IL-6, IL-10, IL-17A, TNF-α, IFN-γ), NLR and QAlb in Anti-NMDAR encephalitis, VE and control groups. All P-values were Bonferroni-corrected. **(A)** Differential analysis of IL-2, IL-4, IL-10, IFN-γ and TNF-α among three groups. **(B)** Differential analysis of IL-6, IL-17A, NLR and QAlb among three groups.

Further, the results indicate that after multiple comparison adjustment using Bonferroni technique, QAlb, IL-4 and IL-17A showed statistically significant differences between the two disease groups **Figure 2**). In anti-NMDA receptor encephalitis, the expression levels of IL-4 and QAlb were lower, whereas the expression level of IL-17A was higher, compared with the VE group. However, cytokines IL-2, IL-6, IL-10, TNF-α, IFN-γ and NLR did not show statistically significant differences between the two groups. And we also compared various indicators between the two disease groups and the control group. As shown in **Figure 2**, the results showed that the indicators with differential expression between the disease groups had statistical differences between the anti-NMDAR group and the control group or between the VE group and the control group.

### 3.3 Establishment of the biomarker evaluation

**Figure 3:**
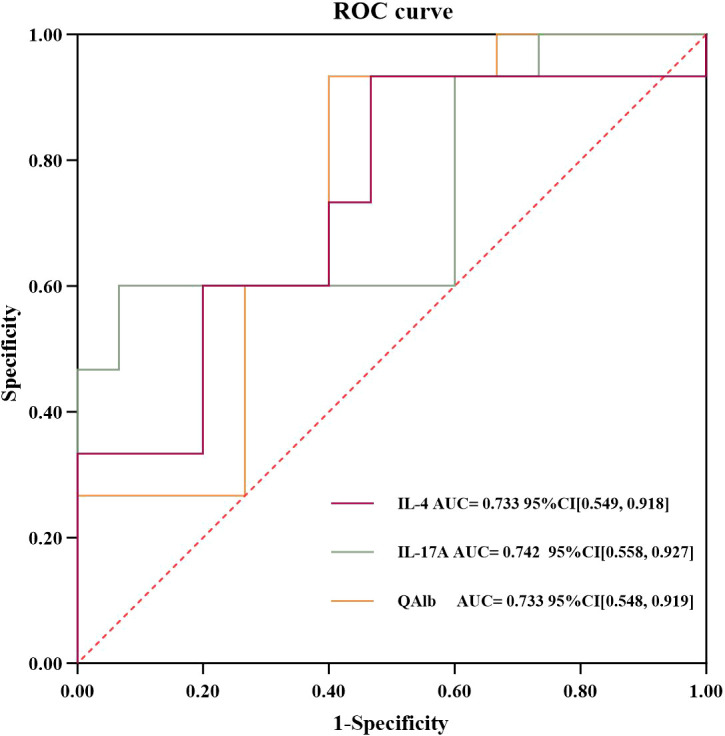
The ROC curve for the significant difference index to distinguish anti-NMDAR encephalitis from VE.

To evaluate the diagnostic performance of the aforementioned differential markers in the two disease groups, ROC curves were plotted. The area under the ROC curve (AUC) was calculated to indicate efficiency and accuracy of identification. The results showed that the AUCs of IL-4, IL-17A and QAlb were 0.733, 0.742 and 0.733 respectively (**Figure 3**); the sensitivities were 0.93, 0.60 and 0.93, respectively; and the specificities were 0.53, 0.93 and 0.60 respectively (**Supplementary Table 2**). The cutoff values of four potential biomarkers of IL-4, IL-17A and QAlb in diagnosing anti-NMDAR encephalitis were 2.8 pg/ml, 1.3 pg/ml and 13.4, respectively. Based on the above data, these biomarkers may serve as potential biomarkers for differentiation of anti-NMDAR encephalitis from VE.

### 3.4 Correlation between various indicators and the severity of the disease

The study evaluated the correlation between the differential expression indicators identified in the two disease groups and the patients ‘hospital stay duration and mRS score at admission, to analyze their relationship with disease severity. The results showed that in the anti-NMDAR encephalitis group, IL-17A and QAlb were positively correlated with the patients’ hospital stay duration and mRS score at admission (P<0.05). In the VE group, IL-4 and QAlb were associated with the patients’ hospital stay duration and disease severity (P<0.05), with IL-4 showing a negative correlation (**Table 3**).

**Table 3:** Correlation analysis of differential expression indicators with the length of hospital stays and the mRS score at admission in patients with anti-NMDAR encephalitis and VE.

Indicators	Anti-NMDAR encephalitis r (p-value)			VE r (p-value)		
	IL4	IL17A	QAlb	IL4	IL17A	QAlb
Hospital stays	-0.478 (0.072)	0.538 (0.038)	0.622 (0.013)	-0.515 (0.049)	-0.277 (0.318)	0.723 (0.002)
mRS	-0.187 (0.504)	0.522 (0.046)	0.783 (0.001)	-0.644 (0.010)	-0.175 (0.533)	0.552 (0.033)

mRS, modified Rankin Scale; anti-NMDAR, anti-N-methyl-D-aspartate receptor; VE, viral encephalitis; QAlb, the CSF/serum albumin ratio.

## 4 Discussion

This study collected data on serum cytokines, QAlb, and NLR from patients diagnosed with anti-NMDAR encephalitis, VE, and the control group with non-inflammatory central nervous system diseases. Comparative analysis revealed statistically significant differences in the levels of IL-4, IL-17 and NLR among the three groups. Furthermore, these biomarkers demonstrated diagnostic relevance for ANTI-NMDAR encephalitis. Among them IL-17A and QAlb were correlated with both the length of hospital stay and mRS scores at admission in anti-NMDAR encephalitis patients. In the VE group, IL-4 and QAlb were similarly associated with these clinical severity indicators. The study further analyzes and discusses the potential clinical implications of these differentially expressed indicators.

IL-4, an anti-inflammatory cytokine pivotal in Th2-mediated immune responses, plays a vital role in modulating neuroinflammation and microglial function (24, 25). While its protective role in mitigating excessive inflammation in VE has been proposed (26), its differential expression across various encephalitis types remains underexplored. Our study aimed to evaluate the potential of IL-4 in distinguishing between anti-NMDAR encephalitis and VE in the early stages.

We observed statistically significant differences in IL-4 levels not only between the disease groups and controls but also between anti-NMDAR encephalitis and VE patients, suggesting its utility as a discriminative biomarker. Furthermore, the negative correlation between IL-4 levels and both hospitalization duration and admission mRS scores specifically in the VE group implies a potential role in restraining excessive inflammation and facilitating neurological recovery following viral infection. The elevated IL-4 in early-stage VE may suppress the production of pro-inflammatory mediators such as TNF-α and IFN-γ, thereby exerting neuroprotective effects and possibly contributing to better outcomes.

The absence of a similar significant correlation in the anti-NMDAR encephalitis group highlights distinct immunopathological mechanisms underlying the two disorders. Whereas VE may involve a more pronounced Th1/Th2 imbalance amenable to IL-4-mediated modulation, anti-NMDAR encephalitis is primarily antibody-driven with a different cytokine profile. This divergence reinforces the notion that IL-4 may serve not only as a diagnostic adjunct but also as an indicator of differential immune endophenotypes in encephalitis.

IL-17A, primarily secreted by Th17 cells, plays a crucial role in the recruitment of immune cells and helps protect the body from bacterial infections (27). Elevated levels of IL-17A have been observed in central nervous system demyelinating diseases (28). Studies have shown that IL-17A can promote inflammation in microglia and the BBB, and experimental administration of IL-17A neutralizing antibodies can prevent the recruitment of neutrophils and the secretion of downstream cytokines, thereby partially alleviating BBB damage (29). Previous research has confirmed that the concentration of IL-17A in the cerebrospinal fluid of patients with acute anti-NMDAR encephalitis is elevated, and the concentration of IL-17A is positively correlated with the severity of the disease, suggesting that IL-17A may be a useful indicator for predicting the condition and prognosis (30). These studies indicate a close relationship between IL-17A levels and anti-NMDAR encephalitis.

Our research found that there are significant differences in IL-17A levels between the two disease groups and between the disease group and the control group, and it is significantly higher in anti-NMDAR encephalitis compared to the VE group and the control group. As a biological marker for early differentiation between anti-NMDAR encephalitis and VE encephalitis, IL-17A levels have diagnostic value. We also confirmed that IL-17A levels are significantly positively correlated with the length of hospital stay and the mRS score at admission in patients with anti-NMDAR encephalitis, indicating a close relationship between serum IL-17A and anti-NMDAR encephalitis, and can serve as an important indicator for assessing the patient’s condition. This finding aligned with the conclusions drawn in previous studies.

QAlb is an established biomarker for evaluating BBB integrity, as increased CSF protein in pathological states primarily consists of albumin due to altered permeability (31). BBB disruption, quantified by QAlb, is frequently associated with disease severity and poor prognosis, as evidenced in conditions like multiple sclerosis and autoimmune encephalitis (32, 33). The extant research indicates that the normal range for QAlb is influenced by gender and age (34). In the context of VE, numerous viruses are known to compromise the BBB and facilitate microinvasions, and elevated QAlb during the acute phase has been linked to severity (35).

Our findings align with this body of evidence, demonstrating significant differences in QAlb between anti-NMDAR encephalitis and VE groups, and positive correlations with hospitalization duration and admission mRS scores in both cohorts. Critically, we observed that QAlb levels were significantly higher in VE patients than in those with anti-NMDAR encephalitis. This suggests that viral pathogens may induce more severe BBB disruption through direct cytopathic effects and intense innate immune activation, compared to the antibody-mediated mechanisms predominant in anti-NMDAR encephalitis. Consequently, QAlb emerges not only as a marker of overall severity but also as a potential discriminator for early differential diagnosis between these two disorders.

### 4.1 Limitation

A key limitation of this study is its relatively small sample size, which may compromise the stability and generalizability of the preliminary findings. As a retrospective pilot study, selection bias could not be completely avoided, even though age-stratified sampling was performed to minimize this bias. Consequently, further validation in larger and prospective cohorts is required to verify the potential diagnostic performance of these candidate biomarkers. A further limitation is that blood samples were collected within 72 hours of admission, without strict standardization of the time interval from disease onset to sample collection. In addition, batch-to-batch variations in laboratory testing were inevitable because samples were not analyzed concurrently. The normal range for QAlb is influenced by age and gender; however, the present study did not adjust for either of these factors. Such analytical variability may also have influenced the present results. Finally, this study lacked external validation, which further restricts the broader generalizability of the findings.

## 5 Conclusion

IL-4, IL-17A and QAlb showed diagnostic potential as a practicable panel of candidate biomarkers for the early differential evaluation and preliminary severity assessment between anti-NMDAR encephalitis and viral encephalitis (VE).These biomarkers represent a relatively cost-effective and readily available laboratory approach that may provide auxiliary information to assist clinicians in the early differential evaluation of these two disorders.

## Data Availability

The raw data supporting the conclusions of this article will be made available by the authors, without undue reservation.
