# Supplementary material for: Blood cytokine levels in anti-NMDAR and viral encephalitis during the early stage of hospitalization: a small-sample exploratory pilot study
Source: Front Immunol. 2026 Apr 1;17:1763289. doi: 10.3389/fimmu.2026.1763289 (PMC13079349; doi:10.3389/fimmu.2026.1763289)
Supplement: Supplementary file 1 [file Table1.docx]

| **Supplementary Table 1.** The baseline balance analysis of key demographic and clinical indicators between included and excluded VE by random sampling method.   \|  \| included VE  (n=15) \| excluded VE  (n=34) \| P value \| \| --- \| --- \| --- \| --- \| \| Age \| 44.73±18.21 \| 45.09±15.01 \| 0.943 ^a^ \| \| Sex（Female） \| 10(66.67%) \| 22(64.71%) \| 0.894 ^b^ \| \| Mental symptom \| 10(66.67%) \| 24(70.59%) \| 1.000 ^b^ \| \| Epilepsy \| 7(46.67%) \| 16(47.06%) \| 0.980 ^b^ \| \| Central hypoventilation \| 4(26.67%) \| 9(26.47%) \| 1.000 ^b^ \| \| Fever \| 5(33.33%) \| 11(32.35%) \| 1.000 ^b^ \| \| Lowering of consciousness \| 8(53.33%) \| 19(55.88%) \| 0.869 ^b^ \| \| Tumour \| 0(0.00%) \| 1(2.94%) \| 1.000 ^b^ \| |
| --- | --- | --- | --- | --- | --- | --- | --- | --- | --- | --- | --- | --- | --- | --- | --- | --- | --- | --- | --- | --- | --- | --- | --- | --- | --- | --- | --- | --- | --- | --- | --- | --- | --- | --- | --- | --- |

Note: ^a^ Student’s t-test; ^b^ Chi-Square test. Data are presented as mean ± SD for normally distributed continuous variables, and as median (maximum, minimum) for non-normally distributed continuous. The enumeration data are n (%).

Abbreviations: VE, viral encephalitis.

**Supplementary Table 2.** ROC analysis results of differentiation between anti-NMDAR encephalitis and VE.

|  | Sensitivity | Specificity | Cutoff | AUC | 95% CI | *P* value |
| --- | --- | --- | --- | --- | --- | --- |
| IL-4 (ng/ml) | 0.93 | 0.53 | 2.8 | 0.733 | 0.549 - 0.918 | 0.03 |
| IL-17A (ng/ml) | 0.60 | 0.93 | 1.3 | 0.742 | 0.558 - 0.927 | 0.02 |
| QAlb | 0.93 | 0.60 | 13.4 | 0.733 | 0.548 - 0.919 | 0.03 |

Abbreviations: ROC, receiver operating characteristics; anti-NMDAR, anti-N-methyl-D-aspartate receptor; VE, viral encephalitis; AUC, the area under the ROC curve; CI, confidence interval; NLR, the neutrophil-to-lymphocyte ratio; QAlb, the CSF/serum albumin ratio.
